# The mutagenic, antimutagenic and antioxidant properties of *Hypericum lydium*

**DOI:** 10.1080/13880209.2016.1242146

**Published:** 2016-12-08

**Authors:** Rukiye Boran, Aysel Ugur

**Affiliations:** a Medical Laboratory Program, Department of Medical Services and Techniques, Vocational School of Health Service, Aksaray University, Aksaray, Turkey;; b Section of Medical Microbiology, Department of Basic Sciences, Faculty of Dentistry, Gazi University, Ankara, Turkey

**Keywords:** Antimutagenicity, free radical damage, natural products

## Abstract

**Context:** There is a growing market demand for *Hypericum* sp., a pharmacologically active plant that has been traditionally used to treat various ailments. However, there have been limited studies on the extract or essential oil of *Hypericum lydium* Boiss (Hypericaceae).

**Objective:** This study investigates for the first time the antioxidant, mutagenic and antimutagenic activity of an ethanol extract of *H. lydium*.

**Material and methods:** Ethanol extract from aerial parts of *H. lydium* harvested from Turkey were tested for this mutagenic and antimutagenic activities (2.0–0.002 mg/plate) using Ames *Salmonella*/microsome test system. 4-Nitro-o-phenylenediamine (4-NPD) (3 μg/plate) for the *Salmonella typhimurium* TA98 and sodium azide (NaN_3_) (8 μg/plate) for the *S. typhimurium* TA100 were used as positive controls. The antioxidant activity, total antioxidant activity and phenolic constituent of the extract (2.0–0.002 mg/mL) was determined by the inhibition of 2,2-diphenyl-1-picrylhydrazyl radical (DPPH), β-carotene-linoleic acid model and by means of Folin–Ciocalteu reagent, respectively.

**Results:** The extract showed no sign of mutagenicity at the tested concentrations (0.002–2.0 mg/mL), and showed concentration-dependent antimutagenic activity against NaN_3_ and 4-NPD ranging from 26.8 to 81.5%. The extract was found to be an efficient scavenger of DPPH (IC_50_ 0.165 ± 0.23 mg/mL) and to inhibit β-carotene-linoleic acid bleaching (IC_50_ 0.39 ± 0.11 mg/mL).

**Discussion and conclusion:** These findings indicate ethanol extract of *H. lydium* to be a safe and effective agent that may be incorporated into new strategies for the prevention of cancer and mutagenesis.

## Introduction

Reactive oxygen species or free radicals such as hydroxyl radical, superoxide radical, hydrogen peroxide and singlet oxygen are believed to induce mutations and inhibit the DNA repair process, inactivating certain tumour-suppressing genes and leading to cancer (Ames 1983; Sanjib 2011). They also damage other cellular components such as proteins, enzymes and membrane lipids. The free radicals generated in the body by various endogenous systems can be removed by its own natural antioxidant defence systems. However, endogenous defences are not completely efficient (Sathuvan et al. 2012). Therefore, the foods and medicinal plants can be an important aspect of body's defence mechanism to protect against free radical damage (Ames 1983; Jayaprakasha et al. 2006). The investigation and discovery of antioxidant, antimutagenic or anticancer properties of plants are of great practical and therapeutic importance (Zahin et al. 2010).

*Hypericum* L. is a relatively large genus of the Hypericaceae family that includes about 484 species of trees, shrubs and herbs (Crockett & Robson 2011). Recently, a growing market niche for *Hyperici herba* products and thus an increasing demand for crude material have resulted in considerable research into the pharmacological activities of the *Hypericum* genus (Odabaş et al. 2009; Cirak et al. 2015). Most of this research has focused on *Hypericum perforatum* L. (Hypericaceae), which is the most common and best known member of this genus (Çırak et al. 2007) and has been certified for marketing as a traditional medicine in many European countries. Although several other *Hypericum* species are also used as traditional medicinal plants to treat a variety of ailments (Russo et al. 2014), comparatively few studies have been reported for other members of the *Hypericum* genus (Çırak et al. 2007).

*Hypericum lydium* Boiss. (Hypericaceae) is a perennial herb that can reach a height of up to 60 cm and has yellow flowers and characteristic translucent glandular dots on the sepal margin (Davis 1988). Known in Turkish as *sancı otu* ("cramp herb") and *mayasıl otu* ("hemorrhoid herb"), *H. lydium* has traditionally been used in folk medicine to treat menstrual disorders, stomach pains, wounds, hemorrhoids and indigestion (Yesilada et al. 1995; Sezik et al. 2001; Altundag & Ozturk 2011).

The present study was designed to examine oxidative damage protecting, mutagenic and antimutagenic activity of ethanol extract from *H. lydium*. To the best of our knowledge, the literature includes only one report on the antioxidant activity of *H. lydium* essential oil and methanol extracts (Şerbetçi et al. 2012), no reports on the antioxidant activity of *H. lydium* ethanol extracts and no reports on the mutagenic or antimutagenic activities of any *H. lydium* oil or extract.

## Materials and methods

### Bacterial strains and chemicals

*Salmonella typhimurium* TA98 and *S. typhimurium* TA100 strains were provided by The American Type Culture Collection, Bacteria Department of Georgetown University, Washington, D.C., USA.

In mutagenicity/antimutagenicity assays, direct acting mutagens NaN_3_ and 4-NPD were obtained from Merck (Darmstadt, Germany) and Sigma-Aldrich (St. Louis, MO), respectively. Other solvents and chemicals including ethanol, chloroform, d-biotin, sodium ammonium phosphate (Na_2_NH_2_PO_4_), l-histidine, magnesium sulfate (MgSO_4_), crystal violet, sodium phosphate (Na_2_HPO_4_), citric acid, d-glucose, potassium phosphate (K_2_HPO_4_), dimethyl sulfoxide (DMSO), butylated hydroxytoluene (BHT), ascorbic acid, DPPH, β-carotene, linoleic acid, Tween 60, Folin-Ciocalteau reagent, gallic acid were also obtained from Sigma, Merck and Difco (Paris, France).

### Plant collection and extract preparation

The aerial parts of *H. lydium* were collected from wild plants growing locality in Adana (37°26′56″N 35°07’′14″W) (Turkey), in June 2012. This plant was authenticated by Dr. Ceylan, and a voucher specimen (ARB-H05) was deposited in the Department of Biology, University of Mugla Sıtkı Kocman, Mugla, Turkey. Thirty grams of *H. lydium* aerial parts were extracted with 350 mL of boiling ethanol in a Soxhlet apparatus for 3 h. Then, the extract was filtered and concentrated by rotary evaporator. The extract was kept at −20 °C and was dissolved in DMSO before use.

### Mutagenicity and antimutagenicity assay

The potential of mutagenic and antimutagenic effects of the ethanol extract of *H. lydium* were evaluated on two histidine-dependant (His^−^) mutant tester strains of *Salmonella typhimurium* TA98 and TA100. Before tests, the tester strains were analyzed for their genetic integrity and spontaneous mutation rate, and cytotoxic doses of the ethanol extract of *H. lydium* were determined by the method of Mortelmans and Zeiger (2000).

The mutagenic effect of the ethanol extract of *H. lydium* was performed using the plate/incorporation procedure as described by Maron and Ames (1983). The positive controls used 4-NPD (3 μg/plate) for the TA98 strain and NaN_3_ (8 μg/plate) for the TA100 strain. The plates were incubated for 48 h at 37 °C, and His^+ ^revertants were counted.

The antimutagenicity against 4-NPD (3 μg/plate for the TA98 strain) and NaN_3_ (8 μg/plate for the TA100 strain) was assayed by incubating with or without 2.0, 0.2, 0.02 and 0.002 mg/plate of ethanol extract of *H. lydium*, using *S. typhimurium* TA98 or TA100 as test organisms. The tube containing DMSO was used to determine spontaneous reversion. After incubating for 72 h, at 37 °C, the His^+ ^revertant colonies were counted. The antimutagenic activity was expressed as a percentage of mutagenic inhibition using the formula:

% inhibition=[1-(T-S)/(C-S)]×100

*T* is the number of revertants in the presence of mutagen and the ethanol extract, *S* is the number of spontaneous revertants and *C* is the number of revertants induced by the mutagen.

### Antioxidant activity assay

#### DPPH radical scavenging activity

The free radical-scavenging activity of the ethanol extract of *H. lydium* was determined using the stable DPPH according to Ebrahimabadi et al. (2010) with a little modification. One mL DPPH (0.2 mM in 95% ethanol) was mixed with 1 mL of the extract of various concentrations in 95% ethanol. After vortexing, the tubes were left in the dark for 30 min at room temperature, after which the absorbance was measured against a blank at 517 nm. BHT and ascorbic acid and were utilized as standard antioxidants. The ability to scavenge DPPH radical was calculated by the following equation:

$$\mathrm{Radical}\;\mathrm{scavenging}\;\mathrm{activity}\;\left(\%\right)=[({\mathrm{Abs}}_{c}-{\mathrm{Abs}}_{s})/{\mathrm{Abs}}_{c}]\times 100$$

Abs_c_ is the absorption of the blank and Abs_s_ is the absorption of the ethanol extract.

#### Inhibition of β-carotene-linoleic acid bleaching assay

The β-carotene bleaching potential of the ethanol extract of *H. lydium* was determined according to by Rauter et al. (2012). In brief, 1 mL of β-carotene (0.5 mg/mL in chloroform) was mixed with 25 μL of linoleic acid and 200 mg of Tween 60. The mixture was shaken and evaporated to remove chloroform. Then, 100 mL of oxygenated distilled water was added to the mixture and agitated. From this emulsion, 2.5 mL transferred into different test tubes containing 0.5 mL of the extract. The initial absorbance of samples was measured after 1 min of vortexing at 470 nm. Samples were incubated for 60 min at 50 °C, and the second absorbance was measured at 470 nm after 1 min of vortexing. BHT and ascorbic acid were used as a control. The measurement was carried out at 30 min intervals. The following formula was used to determine the antioxidant activity (AA%) of the samples:

$$\mathrm{Ln}\;\left(\mathrm{Abs}\right)=\ln\left({\mathrm{Abs}}_{0}\right)+R\times t$$

*R*: the bleaching rate is the slope of ln (Abs) vs. time line, which can be calculated by linear regression, being *t* the time in minutes.

$$\mathrm{AA}\;\left(\%\right)=[({\mathrm{Abs}}_{c}-{\mathrm{Abs}}_{s})/{\mathrm{Abs}}_{c}]\times 100$$

Abs_c_ is the absorption of the blank and Abs_s_ is the absorption of the ethanol extract.

#### Determination of total phenolic content

The total phenolic content was determined according to the Foline Ciocalteu procedure (Singleton et al. 1999). Briefly, 0.1 mL of 0.2 N Folin-Ciocalteau reagent was added to 0.2 mL of extract (1 mg/mL), mixed on a vortex mixer, and after 3 min, the mixture was neutralized with 2 mL of 5% aqueous Na_2_CO_3_ solution. The absorbance of samples was measured at 760 nm after 2 h using Gallic acid as a standard. The concentrations of phenolic compounds were calculated as mg gallic acid equivalents per gram of extract (mg/g GAE extract) using the following equation that was obtained from the standard gallic acid graph: Abs = 0.010 × Gallic acid (μg) + 0.158 (*R* ^2 ^=^ ^0.989)

#### Statistical analysis

All determinations of antioxidant activity were performed in triplicate. Each dose of mutagenic and antimutagenic activity was tested using triplicate plates in two independent experiments. The results are represented as IC_50_ ± standard deviation.

## Results

### Mutagenicity and antimutagenicity assay

For the toxic/highest dose establishing, ethanol extract doses 0.002–2.0 mg/plate were used. Even increasing the concentration of the ethanol extract did not induce any significant increase or decrease in the number of colonies compared with the negative control plates. Thus, for further mutagenicity studies the nontoxic/highest dose selected was 2.0 mg/plate. After exposure to extract, no increase in the number of revertants was observed for two of the strains assayed, TA98 and TA100, at any concentration tested (0.002–2.0 mg/plate) (data not shown).

*H. lydium* ethanol extracts in the nontoxic dose range of 0.002–2.0 mg/plate were also assessed for antimutagenicity *in vitro* against two different types of genotoxic compounds on *S. typhimurium* TA100 and TA98 (Table 1). The maximum inhibition was observed to be 81.5% with a strain of TA100 at the concentration of 2.0 mg/plate, 73.8% at 0.2 mg/plate, 47% at 0.02 mg/plate followed by 42% 0.002 mg/plate. Similarly, the highest inhibition (65%) was obtained by 2.0 mg/plate ethanol extract for TA98 strain.

**Table 1.** Antimutagenicity of the ethanol extract of *H. lydium* against *S. typhimurium* TA98 and TA100.

Number of Revertant Colonies					
		TA98		TA100	
Test items	Concentration	Mean ± S. error	Inhibition (%)	Mean ± S. error	Inhibition (%)
*Negative control*		7 ± 3.6^a^		65 ± 8.6^a^	
*Positive control*					
4-NPD*	3.0 μg/plate	325 ± 32.1			
NaN_3_*	8.0 μg/plate			530 ± 62.4	
Ethanol extract	2.0 mg/plate	114 ± 13.3	65	98 ± 15.1	81.5
	0.2 mg/plate	201 ± 16.1	38.2	139 ± 34.5	73.8
	0.02 mg/plate	207 ± 3.6	36.3	281 ± 47.5	47
	0.002 mg/plate	238 ± 25.1	26.8	307 ± 37.7	42

* 4-NPD and NaN_3_ were used as positive controls for S*. typhimurium* TA98 and TA100 strains, respectively.

a Values expressed are means ± S.D. of three replications. Regression analysis for mutagenicity inhibition (%) and ethanol extract concentrations (log values) was performed using Microsoft Excel.

### Antioxidant activity

An ethanol extract of *H. lydium* was subjected to screening for possible antioxidant activity with DPPH free-radical scavenging and β-carotene-linoleic acid bleaching assays (Table 2). According to the DPPH assay results, IC_50_ values of the *H. lydium* extract, ascorbic acid and BHT were 0.165 ± 0.23 mg/mL, 0.01 ± 0.03 mg/mL and 0.184 ± 0.01 mg/mL, respectively. The total AA increased with an increase in the concentration of the extracts. The IC_50_ of the extract was 0.39 ± 0.11 mg/mL, whereas those of ascorbic acid and BHT were 0.020 ± 0.2 mg/mL and 0.05 ± 0.012 mg/mL, respectively. The total phenolic content was 135 ± 1.11 mg Gallic acid equivalent/g extract.

**Table 2.** Radical scavenging activity, total antioxidant activity and total phenolic content of the *H. lydium* extract.

	Test systems		
Samples	DPPH^a^ IC_50_ (mg/mL)	β-carotene–linoleic acid^a^ IC_50_ (mg/mL)	Total phenols GAE/(mg/mL)
*Hypericum lydium*	0.165 ± 0.23	0.39 ± 0.11	135 ± 1.11
BHT^b^	0.184 ± 0.01	0.05 ± 0.012	Ns^c^
Ascorbic acid^b^	0.01 ± 0.03	0.020 ± 0.2	Ns^c^

a IC_50_ values represent the means ± standard deviation of three parallel measurements (*p* < 0.05).

b Reference compounds.

c Not studied.

## Discussion

Ames *Salmonella*/microsome test results showed that the ethanol extract of *H. lydium* were neither toxic nor mutagenic to the bacteria at the tested concentrations (data not shown). The Ames test is the most commonly used test for determining mutagenicity and carcinogenicity and utilizes strains of *S. typhimurium* (TA100 and TA98) that are specifically modified (His^−^) to enhance their sensitivity to mutagenicity testing (Ames et al. 1975). In order to be considered mutagenic, a chemical agent must induce a number of revertant colonies at least twice that of the number of colonies appearing in a negative control plate.

In this study, the ethanol extract of *H. lydium* was observed not to exhibit positive mutagenicity in the Ames test at four different concentrations ranging between 0.002 and 2.0 mg/plate. Mutagens are not only related in carcinogenesis and genotoxicity but also in the start and pathogenesis of several chronic degenerative diseases. One of the best ways to minimize the harmful action of mutagens is by the use of natural antimutagens (Satish et al. 2013).

To assess the antimutagenic effects of the *H. lydium* ethanol extracts, induction or suppression of revertant colonies were examined in the same tester strains. All four doses of ethanol extract tested were found to effectively inhibit mutagenicity of 4-NDP and NaN_3_, with a linear dose-response relationship in antimutagenic activity observed with both mutagens (Table 1). A strong antimutagenic effect (65%) was observed with TA98 at a 2.0 mg/plate concentration, whereas moderate antimutagenic effects were observed at the other concentrations tested. Strong antimutagenic effects ranging from 42 to 81.5% were also observed with TA 100 for all four concentrations tested. Reductions were observed in both base-substitution mutagenicity induced by NaN_3_ and frameshift mutagenicity induced by 4-NDP, suggesting that *H. lydium* extract acts through different mechanisms. The literature contains no previous reports on the antimutagenic effects of *H. lydium*.

Previous studies have reported *H. lydium* to contain chlorogenic acid, quercetin, rutin, hyperoside, quercitrin, apigenin-7-*O*-glucoside, hypericin and pseudohypericin (Çırak 2006; Çırak et al. 2007). Hypericin and pseudohypericin are naturally occurring red pigments that have been reported to exhibit important biological activities (Guedes & Eriksson 2005). Research has suggested that many of the pharmacological activities of *Hypericum* extracts can be attributed to their hypericin contents (Barnes et al. 2001).

In addition, the ethanol extract of the *H. lydium* was investigated for antioxidant activity with DPPH free-radical scavenging and β-carotene-linoleic acid bleaching assays (Table 2). Antioxidants are believed to affect DPPH through their hydrogen-donating activity (Baumann et al. 1979). As a lipophilic radical, DPPH readily accepts electrons from antioxidant compounds, resulting in a change in colour from purple to yellow that is detectable at 517 nm (Inbathamizh et al. 2013). In the present study, a change in colour from purple to yellow observed in the ethanol extract of *H. lydium* indicated a high level of radical-scavenging activity. IC_50_ values of the *H. lydium* extract, ascorbic acid and BHT were 0.165 ± 0.23 mg/mL, 0.01 ± 0.03 mg/mL and 0.184 ± 0.01 mg/mL, respectively. According to the DPPH assay results, it was observed that extract presented higher antioxidant activity than BHT. Numerous studies have reported on the antioxidant activities of *Hypericum* species (Hernandez et al. 2010; Zorzetto et al. 2015); however, only one study has reported on the antioxidant activity of *H. lydium* (Şerbetçi et al. 2012), and that study examined the activity of methanol extracts of the plant. Methanol extracts of fruits and flowers were reported to have similar EC_50_ values (1.03 ± 0.13 mg/mL and 0.99 ± 0.25 mg/mL, respectively), both of which were lower than the values found for the ethanol extract in the present study.

The β-carotene bleaching assay is widely used to measure the antioxidant activity of bioactive compounds. In this study, the β-carotene-linoleic acid assay was used to measure the antioxidant activity of *H. lydium* ethanol extracts and compare it with that of BHT and ascorbic acid. The results found the IC_50_ values of the ethanol extract, ascorbic acid and BHT to be 0.39 ± 0.11 mg/mL, 0.020 ± 0.2 mg/mL and 0.05 ± 0.012 mg/mL, respectively (Table 2).

This study measured the total phenolic contents of an *H. lydium* ethanol extract using Folin–Ciocalteu’s method, which presents total phenolic contents as gallic acid equivalents using a standard curve plotted using the average absorbance values of two independent sets of data against concentrations of gallic acid in μg/mL. The present study found *H. lydium* ethanol extract to contain a high level of total phenolic compounds (135 ± 1.11 mg GAE/g extract). The phenolic compounds in plants represent the main class of natural antioxidants (Tajkarimi et al. 2010). A previous study by Şerbetçi et al. (2012) reported GAE of methanol extracts of *H. lydium* fruits and flowers per gram of dry weight to be 1.61 ± 0.17 and 1.92 ± 0.22 mg, respectively. Considering the findings of the present study as well as previous studies regarding the phenolic constituent profiles of other *Hypericum* species (Çırak et al. 2011; Zorzetto et al. 2015), the *Hypericum* genus appears to provide a potential source of antioxidants.

## Conclusion

Research over the past few years has revealed that mutation has a key role in carcinogenesis. In this study, *H. lydium* ethanol extract did not induce any mutations on the Ames *Salmonella*/microsome test system. In addition, the present study has shown for the first time that *H. lydium* ethanol extract is a promising source for its antioxidant and antimutagenic compounds. These results indicate that *H. lydium* may be considered to be a safe and useful agent for the prevention of cancer and mutations.
